# Supplementary material for: Comparison between Three Different Techniques for the Detection of EGFR Mutations in Liquid Biopsies of Patients with Advanced Stage Lung Adenocarcinoma
Source: Int J Mol Sci. 2023 Mar 29;24(7):6410. doi: 10.3390/ijms24076410 (PMC10094170; doi:10.3390/ijms24076410)
Supplement: Supplementary file 1 [file ijms-24-06410-s001.zip › ijms-2177723-supplementary.pdf]

Supplementary Table S1. Concordance in *EGFR* mutation detection between Therascreen, Idylla and NGS HotSpot Cancer panel assays among the 54 blood samples analyzed.

**N**	**Sample**	**Therascreen T790M**	**Idylla T790M**	**NGS T790M**	**Therascreen Del EX19**	**Idylla Del EX 19**	**NGS Del EX 19**	**Therascreen L858R**	**Idylla L858R**	**NGS L858R**
1	TRF				POS	POS	POS			
2	DSM				POS	POS	POS			
3	GAM				POS	POS	POS			
4	MSN-2				POS	POS	POS			
5	ARG	POS	POS	POS	POS	POS	POS			
6	VCV	POS	POS	POS	POS	POS	POS			
7	PLG	POS	POS	POS						
8	SRL	POS	POS	POS				POS	POS	POS
9	AAC							POS	POS	POS
10	LBG							POS	POS	POS
11	LSL-1							POS	POS	POS
12	LSL-2							POS	POS	POS
13	AMM									
14	APG									
15	GGA									
16	GSA									
17	MMT									
18	MSA									
19	MSN-1									
20	MOA-2									
21	PMA									
22	PMS									
23	PDA									
24	RML									
25	SLG									
26	CMM									
27	DTA-1									
28	DTA-2									
29	DLR									
30	BLB									
31	CSC									
32	CRG									
33	GRA									
34	MLM									
35	MMG									
36	MLA									
37	MRG									
38	NNA									
**39**	**GGC**							**POS**	**POS**	
**40**	**PLG**								**POS**	
**41**	**VCF**							**POS**	**POS**	
**42**	**ASG-1**				**POS**	**POS**				
**43**	**ASG-2**				**POS**					
**44**	**DAM**				**POS**					
**45**	**DSA**				**POS**		**POS**			
**46**	**MSN-3**				**POS**	**POS**				
**47**	**NDA**				**POS**					
**48**	**PDG**				**POS**	**POS**				
**49**	**TRE**				**POS**	**POS**				
**50**	**BRA**			**POS**	**POS**	**POS**	**POS**			
**51**	**CHG**	**POS**	**POS**		**POS**	**POS**	**POS**			
**52**	**MOA-1**	**POS**	**POS**		**POS**	**POS**	**POS**			
**53**	**PMC**	**POS**	**POS**		**POS**	**POS**	**POS**			
**54**	**SCL**	**POS**	**POS**		**POS**	**POS**	**POS**			

Supplementary Table S2. *EGFR* mutations detected by Therascreen EGFR Plasma RGQ PCR Kit

EGFR Exon	***COSMIC ID***	***Nucleotide Changes***	***Amino acidic Changes***
19	12422	c.2238_2248delinsGC	p.L747_A750>P
19	6218	c.2239_2247del9	p.L747_E749del
19	12383	c.2239_2251>C	p.L747_T751>P
19	6210	c.2240_2251del12	p.L747_T751>S
19	13551	c.2235_2252>AAT	p.E746_T751>I
19	6223	c.2235_2249del15	p.E746_A750del
19	6225	c.2236_2250del15	p.E746_A750del
19	12678	c.2237_2251del15	p.E746_T751>A
19	6254	c.2239_2253del15	p.L747_T751del
19	12369	c.2240_2254del15	p.L747_T751del
19	12387	c.2239_2258delinsCA	p.L747_P753>Q
19	12370	c.2240_2257del18	p.L747_P753>S
19	12728	c.2236_2253del18	p.E746_T751del
19	12367	c.2237_2254del18	p.E746_S752>A
19	12384	c.2237_2255delinsT	p.E746_S752>V
19	6220	c.2238_2255del18	p.E746_S752>D
19	6255	c.2239_2256del18	p.L747_S752del
19	12419	c.2238_2252>GCA	p.L747_T751>Q
19	12382	c.2239_2248TTAAGAGAAG>C	p.L747_A750>P
20	6240	c.2369C>T	p.T790M
21	6224	c.2573T>G	p.L858R

Supplementary Table S3. *EGFR* mutations detected by Idylla™ ctEGFR Mutation Assay

EGFR Exon	***COSMIC ID***	***Nucleotide Changes***	***Amino acidic Changes***
18	18441	c.2154_2155delinsTT	p.G719C
18	6252	c.2155G>A	p.G719S
18	6253	c.2155G>T	p.G719C
18	6239	c.2156G>C	p.G719A
19	No Cosmic	c.2239_2248delinsC	p.L747_A750>P
19	12370	c.2240_2248del	p.L747_A750delinsS
19	12422	c.2238_2248delinsGC	p.L747_A750>P
19	6218	c.2239_2247del9	p.L747_E749del
19	12383	c.2239_2251>C	p.L747_T751>P
19	6210	c.2240_2251del12	p.L747_T751>S
19	85798	c.2230_2249delinsGTCAA	p.I744_A750>VL
19	1190791	c.2234_2248del	p.K745_A750delinsT
19	13549	c.2235_2251delinsAG	p.E746_T751>A
19	No Cosmic	c.2236_2253delinsCAA	p.E746_T751>Q
19	No Cosmic	c.2236_2253delinsCTA	p.E746_T751>L
19	No Cosmic	c.2237_2253delinsTA	p.E746_T751>V
19	12386	c.2237_2252delinsT	p.E746_T751>V
19	13551	c.2235_2252>AAT	p.E746_T751>I
19	6223	c.2235_2249del15	p.E746_A750del
19	6225	c.2236_2250del15	p.E746_A750del
19	12678	c.2237_2251del15	p.E746_T751>A
19	6254	c.2239_2253del15	p.L747_T751del
19	12369	c.2240_2254del15	p.L747_T751del
19	18426	c.2237_2256delinsTC	p.E746_S752>V
19	No Cosmic	c.2235_2255delinsGGT	p.E746_S752>V
19	No Cosmic	c.2237_2256delinsTT	p.E746_S752>V
19	12387	c.2239_2258delinsCA	p.L747_P753>Q
19	12370	c.2240_2257del18	p.L747_P753>S
19	12728	c.2236_2253del18	p.E746_T751del
19	12367	c.2237_2254del18	p.E746_S752>A
19	12384	c.2237_2255delinsT	p.E746_S752>V
19	6220	c.2238_2255del18	p.E746_S752>D
19	6255	c.2239_2256del18	p.L747_S752del
19	255211	c.2238_2258del	p.L747_P753del
19	133189	c.2236_2256del21	p.E746_S752del
19	13556	c.2253_2276del24	p.S752_I759del
20	No Cosmic	c.2307_2308delinsGCCAGCGTG	p.D769_D770insASV
20	No Cosmic	c.2309_2310delinsCCAGCGTGGAT	p.D769_D770insASV
20	6241	c.2303G>T	p.S768I
20	12378	c.2310_2311insGGT	p.D770_N771insG
20	48922	c.2311_2312insGCGTGGACA	p.D770_N771insSVD
20	12377	c.2319_2320insCAC	p.H773_V774insH
20	6240	c.2369C>T	p.T790M
21	133630	c.2573_2574TG>GA	p.L858R
21	12429	c.2573_2574TG>GT	p.L858R
21	6213	c.2582T>A	p.L861Q
21	6224	c.2573T>G	p.L858R

Supplementary Table S4. *EGFR* mutations identified by Ion AmpliSeq™ Cancer Hotspot Panel v2

EGFR Exon	*COSMIC ID*	*Nucleotide Changes*	**Amino acidic Changes**
3	21683	c.323G>A	p.R108K
7	21686	c.865G>A	p.A289T
7	21685	c.866C>A	p.A289D
7	21687	c.866C>T	p.A289V
15	21689	c.1787C>T	p.P596L
15	21690	c.1793G>T	p.G598V
18	41905	c.2092G>A	p.A698T
18	28508	c.2104G>T	p.A702S
18	28511	c.2108T>C	p.L703P
18	12988	c.2125G>A	p.E709K
18	12428	c.2125_2127GAA>CAT	p.E709H
18	12371	c.2126A>T	p.E709V
18	13009	c.2126A>G	p.E709G
18	13427	c.2126A>C	p.E709A
18	41603	c.2134T>C	p.F712L
18	28601	c.2135T>C	p.F712S
18	18441	c.2154_2155GG>TT	p.G719C
18	6252	c.2155G>A	p.G719S
18	6253	c.2155G>T	p.G719C
18	18425	c.2156G>A	p.G719D
18	6239	c.2156G>C	p.G719A
18	12373	c.2159C>T	p.S720F
18	22992	c.2161G>A	p.G721S
18	28510	c.2162G>C	p.G721A
18	13979	c.2170G>A	p.G724S
19	13180	c.2188C>T	p.L730F
19	13432	c.2193G>A	p.W731*
19	53194	c.2197C>T	p.P733S
19	13181	c.2198C>T	p.P733L
19	18419	c.2200G>A	p.E734K
19	13182	c.2203G>A	p.G735S
19	27041	c.2213T>G	p.V738G
19	17570	c.2222C>T	p.P741L
19	13183	c.2225T>C	p.V742A
19	26509	c.2227G>A	p.A743T
19	26038	c.2233_2247del15	p.K745_E749del
19	14243	c.2234A>G	p.K745R
19	28517	c.2235_2246del12	p.E746_E749del
19	6223	c.2235_2249del15	p.E746_A750del
19	24869	c.2235_2252del18	p.E746_T751del
19	13184	c.2236G>A	p.E746K
19	6225	c.2236_2250del15	p.E746_A750del
19	12728	c.2236_2253del18	p.E746_T751del
19	133189	c.2236_2256del21	p.E746_S752del
19	12678	c.2237_2251del15	p.E746_T751>A
19	12386	c.2237_2252>T	p.E746_T751>V
19	12367	c.2237_2254del18	p.E746_S752>A
19	12384	c.2237_2255>T	p.E746_S752>V
19	23571	c.2238_2252del15	p.L747_T751del
19	6218	c.2239_2247del9	p.L747_E749del
19	12382	c.2239_2248TTAAGAGAAG>C	p.L747_A750>P
19	12383	c.2239_2251>C	p.L747_T751>P
19	6254	c.2239_2253del15	p.L747_T751del
19	6255	c.2239_2256del18	p.L747_S752del
19	133197	c.2239_2257>T	p.L747_P753>S
19	26704	c.2240T>C	p.L747S
19	6210	c.2240_2251del12	p.L747_T751>S
19	12369	c.2240_2254del15	p.L747_T751del
19	12370	c.2240_2257del18	p.L747_P753>S
19	18442	c.2241_2244AAGA>CCCG	p.L747_R748>FP
19	6219	c.2248G>C	p.A750P
19	13185	c.2252C>T	p.T751I

19	133207	c.2252_2275del24	p.T751_I759del
19	96856	c.2252_2276>A	p.T751_I759>N
19	13556	c.2253_2276del24	p.S752_I759del
19	29274	c.2254T>C	p.S752P
19	6256	c.2254_2277del24	p.S752_I759del
19	13186	c.2255C>A	p.S752Y
19	6268	c.2257C>T	p.P753S
19	85993	c.2260A>G	p.K754E
19	13188	c.2281G>A	p.D761N
19	21984	c.2281G>T	p.D761Y
19	27042	c.2282A>G	p.D761G
20	28603	c.2293G>A	p.V765M
20	26445	c.2300C>T	p.A767V
20	6241	c.2303G>T	p.S768I
20	6242	c.2305G>T	p.V769L
20	12376	c.2307_2308ins9	p.V769_D770insASV
20	14068	c.2308G>A	p.D770N
20	12427	c.2308_2309insGTT	p.D770>GY
20	12378	c.2310_2311insGGT	p.D770_N771insG
20	13003	c.2310_2311insAAC	p.D770_N771insN
20	13004	c.2310_2311insGGC	p.D770_N771insG
20	48922	c.2311_2312insGCGTGGACA	p.D770_N771insSVD
20	13428	c.2311_2312ins9	p.D770_N771insSVD
20	13005	c.2318A>T	p.H773L
20	13433	c.2318A>G	p.H773R
20	12377	c.2319_2320insCAC	p.H773_V774insH
20	12381	c.2319_2320ins9	p.H773_V774insNPH
20	13006	c.2320G>A	p.V774M
20	22954	c.2324G>A	p.C775Y
20	6226	c.2326C>T	p.R776C
20	22940	c.2327G>A	p.R776H
20	13007	c.2335_2336GG>TT	p.G779F
20	28513	c.2350T>C	p.S784P
20	13189	c.2351C>T	p.S784F
20	27110	c.2356G>A	p.V786M
20	6240	c.2369C>T	p.T790M
20	13190	c.2375T>C	p.L792P
20	12986	c.2429G>A	p.G810D
20	28610	c.2441T>C	p.L814P
20	13400	c.2457G>A	p.V819V
20	41663	c.2462T>C	p.I821T
21	12366	c.2572C>A	p.L858M
21	26129	c.2572C>T	p.L858L
21	13553	c.2572_2573CT>AG	p.L858R
21	6224	c.2573T>G	p.L858R
21	12429	c.2573_2574TG>GT	p.L858R
21	12675	c.2575G>A	p.A859T
21	12374	c.2582T>G	p.L861R
21	6213	c.2582T>A	p.L861Q
21	14070	c.2588G>A	p.G863D
21	13197	c.2590G>A	p.A864T
21	28607	c.2603A>G	p.E868G
21	53292	c.2608C>T	p.H870Y
21	33725	c.2609A>G	p.H870R
21	28605	c.2611G>A	p.A871T
21	13008	c.2612C>G	p.A871G
21	13199	c.2618G>A	p.G873E
21	26438	c.2620G>A	p.G874S
